# Lipids and genes: Regulatory roles of lipids in RNA expression

**DOI:** 10.1002/ctm2.863

**Published:** 2022-05-19

**Authors:** Xiangdong Wang, Xianlin Han, Charles A. Powell

**Affiliations:** ^1^ Department of Pulmonary and Critical Care Medicine Zhongshan Hospital Fudan University Shanghai Medical College Shanghai China; ^2^ Shanghai Institute of Clinical Bioinformatics Shanghai China; ^3^ Shanghai Engineering Research for AI Technology for Cardiopulmonary Diseases Shanghai China; ^4^ Department of Medicine ‐ Diabetes University of Texas Health Science Center at San Antonio San Antonio Texas USA; ^5^ Division of Pulmonary Critical Care and Sleep Medicine Icahn School of Medicine at Mount Sinai New York New York USA

**Keywords:** disease, genes, lipid metabolism, lipids, lipotranscriptome, nuclear lipid droplets

## Abstract

Nuclear lipid metabolism and metabolites play important roles in regulation of lipid–lipid, lipid–gene, lipid–chromatin, lipid–membrane and lipid–protein interactions to maintain the nuclear microenvironment, three‐dimensional chromatin architecture, gene expression and transcription and biological function. This Editorial highlights the value of lipid–gene interaction in the identification and development of biomarkers and targets and emphasizes the significance of inter‐regulation between lipids and genes in innovation and application of precise therapies for patients. Regulatory functions of nuclear lipid dynamics and biophysics modify transcriptomic expression, and modification (lipotranscriptome) can be a new approach for discovery and development of disease‐specific diagnoses and therapies, although there are several challenges to be overcome. Better understanding of how lipid‐based changes of nuclear functions and transcriptomic profiles modify clinical phenomes will provide new insights to understand molecular mechanisms of diseases and to develop spatiotemporal molecular medicine diagnostics and therapeutics.

1

Lipid metabolism and metabolites play an irreplaceable role in maintenance of cell biology and function. The rapid development of methodologies allows us to provide new insights for monitoring the dynamics of lipid metabolism, associated gene transcription, specific protein activation and molecular network regulation. Lipid metabolism in diseases is an important part of clinical and translational medicine and has been selected as a thematic issue. Dysregulation of lipid metabolism contributes to mitochondrial injury and inter‐organelle dysrfunction,^1^, ^2^ imbalance between cell proliferation and cell death^3^, ^4^ and acceleration of epithelial‐mesenchymal transition and cancer cell metastasis.^5^, ^6^ Growing evidence demonstrates that metabolites and molecules generated during disease‐associated lipid metabolism can be an important source to identify and develop new diagnostic biomarkers and therapeutic targets.^1^, ^2^, ^7^, ^8^, ^9^ The clinical lipidomics platform integrates lipidomic profiles with clinical phenomes to uncover disease stage‐, severity‐, duration‐ and type‐specific biomarkers.^10^, ^11^ The interaction between lipids and genes plays critical roles in expression and activation of gene‐specific lipid proteins and enzymes and lipid metabolism‐associated genes, as well as in multi‐directional regulations. This Editorial highlights the value of lipid‐gene interaction in the identification and development of biomarkers and targets and emphasizes the significance of inter‐regulation between lipids and genes in innovation and application of precise therapies for patients.

The interaction between lipids and genes within the nucleus influences functions of the genome without changing the nucleotide sequence (lipotranscriptome), as an epigenetic modification (epitranscriptome). Appropriate expression, transcription and translation of mRNA require proper quality and quantity of multiple factors and formulation of fluid dynamics within the nucleus to regulate and balance the interaction and binding between molecules. Specifically, the amount and function of lipids localized in nuclear membranes, nucleoli, nuclear matrix and chromatin contribute to the construction of inner nuclear membrane (INM) and the function of RNA–DNA activities.^12^ Nuclear lipids include nuclear lipid microdomains and lipid droplets that vary amongst intra‐nuclear localizations and cell types and have various membrane biophysical functions that influence permeability, fluidity, lipid mobility and domain formation. Chromatin‐lipid interaction and chromatin lipid metabolism regulate transcriptional and replicative chromatin activities, linking with the signal transduction pathway functions. Lipid metabolism occurs in the INM, indirectly influencing the three‐dimensional genome, while phospholipid synthesis in the outer nuclear membrane maintains the nuclear integrity. The INM‐promoted lipid storage contributes to the synthesis of nuclear lipid droplets (NLDs) through Seipin‐dependent membrane bridges, genetic circuits for NLD synthesis, and interaction between nuclei and endoplasmic reticulum by sequestration of transcription factors.^13^, ^14^ Abnormalities of gene‐lipid interactions and nuclear lipid metabolism cause dysfunction of genome regulation, transcriptomic expression, nuclear receptor activation that correlate with cell phenome and death, although the exact mechanisms need to be further explored.

Lipid elements and metabolism regulate gene expression, DNA duplication and transcription directly by lipid binding to nuclear transcriptional proteins and receptors, and/or indirectly by modulating the intra‐nuclear microenvironment, membrane biophysics or other intra‐cellular mechanisms. In patterns distinct from those induced by inflammatory mediators and pathogens,^15^ administration of external lipids or increase/reduction of intra‐cellular production/transport can change transcriptomic profiles and expression, although the exact mechanisms remain unclear. Within the fat nucleosome, Lipids directly interact with chromatin though multiple binding sites between, affect chromatin structure and condensed regions, and alter gene expression and cell phenotypes.^16^ For example, cholesterol is required to maintain chromatin organization and remodeling and direct transcriptional functions by interactions with a conserved cholesterol interaction motif and promoter region of Wilms tumor 1 transcriptional corepressor gene, as a gene‐specific target of cholesterol.^17^ Other metabolites can directly interact with chromatin as main substrates or cofactors of chromatin‐modifying enzymes, regulate properties and functions of uncommon regulatory molecules from lipid intermediates and take part in atypical enzymatic and non‐enzymatic chromatin modifications.

Another mechanism by which lipids directly act with RNA is through lipid membrane binding with RNA through the G‐quadruplex formation and riboregulation of guanine residues in short RNAs, which are dependent upon RNA nucleotide content, base pairing and length.^18^ The lipid‐RNA interaction and modification of ribozyme activity occur on the lipid membrane as synthetic riboswitches and RNA‐based lipid biosensors. Different from lipid‐DNA complexes, the lipid‐transfer RNA (tRNA) complexes play critical roles in maintenance of tRNA folding status, aggregation, stability and condensation and are dependent on lipid metabolite chemical properties, concentrations, binding locations and action duration. In addition, nuclear membrane lipid biosynthesis and metabolism participate in the process of genome protection by coordinating the remodeling of the nuclear envelope. Reshaping of the nuclear lipid membrane facilitates forming and fusing of nuclear‐pore complexes, indirectly contributing to gene expression and gene construction.

NLDs are important for lipid appearance, transformation, storage and function in the nuclei and contribute to the INM formation and the maintenance of nuclear homeostasis and microenvironment. Different from cytoplasmic lipid droplets responsible for structural and functional connection between intra‐cellular organelles, NLDs separate the nuclei from other organelles to segregate nuclear energy and function, although molecular mechanisms of NLD formation and origination vary among cell types.^13^, ^19^ Multiple factors are dependently or independently involved in NLD synthesis and lipolysis, including lipoprotein precursors, triglyceride synthesis enzymes, mTOR signaling, lipin‐1, seipin, fatty acid‐binding proteins and choline kinases alpha. Although intra‐cellular lipid droplets have been found to be associated with multiple diseases and metabolic disorders, the precise roles of NLDs in pathogeneses and pathophysiological processes remain unclear.

Advances in the understanding of the clinical and translational medicine roles of gene‐lipid interaction require further definition of the biochemical structures and stability of small lipid elements and metabolites during biological processes and in response to microenvironmental perturbations. It will be important to define the binding sites of lipid elements to nucleosomes or chromatins, for example, molecular size and structure, affinitive specificity, regulators and function‐dependent biophysics, so as to advance the discovery and development of new therapies. It will be important to clarify whether lipid‐chromatin interactions‐associated processes and participants are measurable and are disease phenome‐specific. It is still unclear if transcriptional factors and regulators involving cytoplasmic lipid metabolism, lipid droplet formation and organelle membrane biophysics have a role in the structural and functional dynamics of NLDs. There is an urgent need to characterize direct and indirect effects of NLDs in gene expression and transcription, biological associations between nuclear and cytoplasmic lipid metabolism and droplet biochemical properties and intercommunication between nuclear and cytoplasmic lipid microenvironments. Advances will require technical advances to develop simple and repeatable methods that are sufficiently sensitive to dynamically monitor changes of nuclear lipid contents, precise enough to spatiotemporally track the heterogeneity of the lipid atlas within nuclei and between nuclei and cytoplasm and sophisticated enough to define the interaction and links between nuclear lipidomics and transcriptomics. To translate nuclear lipid systems biology and lipotranscriptomics research into clinical practice, it will be important to better understand the relationship of nuclear lipid structure and function with disease classification, stages, phases, phenomes, responses to interventions and prognoses.

In conclusion, nuclear lipid metabolism and metabolites play important roles in regulation of lipid–lipid, lipid–gene, lipid–chromatin, lipid‐membrane and lipid‐protein interactions to maintain the nuclear microenvironment, three‐dimensional chromatin architecture, gene expression and transcription and biological function. Regulatory functions of nuclear lipid dynamics and biophysics modify transcriptomic expression, and modification (lipotranscriptome) can be a new approach for discovery and development of disease‐specific diagnoses and therapies, although there are several challenges to be overcome. Better understanding of how lipid‐based changes of nuclear functions and transcriptomic profiles modify clinical phenomes will provide new insights to understand molecular mechanisms of diseases and to develop spatiotemporal molecular medicine diagnostics and therapeutics.

## CONFLICT OF INTEREST

The authors declare that there is no conflict of interest that could be perceived as prejudicing the impartiality of the research reported.
